# Prediction of alcohol intake patterns with olfactory and gustatory brain connectivity networks

**DOI:** 10.1038/s41386-025-02058-7

**Published:** 2025-02-17

**Authors:** Khushbu Agarwal, Shefali Chaudhary, Dardo Tomasi, Nora D. Volkow, Paule V. Joseph

**Affiliations:** 1 Section of Sensory Science and Metabolism, National Institute on Alcohol Abuse and Alcoholism, National Institutes of Health, Department of Health and Human Services, Bethesda, MD 20892 USA; 2 Department of Psychiatry, Yale University School of Medicine, New Haven, CT 06519 USA; 3 Laboratory of Neuroimaging, National Institute on Alcohol Abuse and Alcoholism, National Institutes of Health, Department of Health and Human Services, Bethesda, MD 20892 USA; 4 National Smell and Taste Center, National Institutes of Health, Department of Health and Human Services, Bethesda, MD 20892 USA; 5 National Institute on Deafness and Other Communication Disorders, National Institutes of Health, Department of Health and Human Services, Bethesda, MD 20892 USA; 6 Present Address: National Brain Research Center, NH-8, Manesar, Gurugram, Haryana 122052 India

**Keywords:** Outcomes research, Neuronal physiology

## Abstract

Craving in alcohol drinkers is often triggered by chemosensory cues, such as taste and smell, which are linked to brain network connectivity. This study aimed to investigate whether these brain connectivity patterns could predict alcohol intake in young adults. Resting-state fMRI data were obtained from the Human Connectome Project (HCP) Young Adult cohort, comprising 1003 participants. Functional connectomes generated from 100 independent components were analyzed, identifying significant connections correlated with taste and odor scores after applying a false discovery rate (FDR) correction using the Benjamini-Hochberg (BH) method. These significant connections were then utilized as predictors in general linear models for various alcohol intake metrics. The models were validated in an independent sample to assess their accuracy. The training sample (*n* = 702) and the validation sample (*n* = 117) showed no significant demographic differences. Out of 742 possible connections, 41 related to odor and 25 related to taste passed the significance threshold (*P* < 0.05) after FDR-BH correction. Notable predictors included visual-visual connectivity (node32-node13: β = 0.028, *P* = 0.02) for wine consumption and connectivity between the ventral attention network (VAN) and the frontal parietal/caudate nucleus (FP/CN) (node27-node9: β = −0.31, *P* = 0.04) for total alcohol intake in the past-week and maximum number of drinks per day in the past-year. The predictive models demonstrated strong accuracy, with root mean square error (RMSE) values of 5.15 for odor-related models and 5.14 for taste-related models. The F1 scores were 0.74 for the odor model and 0.71 for the taste model, indicating reliable performance. These findings suggest that specific patterns of brain connectivity associated with taste and olfactory perception may serve as predictors of alcohol consumption behaviors in young adults. Our study highlight the need for longitudinal research to evaluate the potential of taste- and smell-related brain connectivity patterns for early screening and targeted interventions, as well as their role in personalized treatment strategies for individuals at risk of AUD.

## Introduction

An estimated 140,000 deaths (approximately 97,000 male deaths and 43,000 female deaths) are annually attributed to chronic alcohol use in United States [1, 2] and young adults are at a greater risk for alcohol use disorder (AUD) [3, 4]. Alcohol craving is one of the main mechanisms underlying alcohol consumption [5] and thus serves as an important determinant of AUD [6]. Among the many sensory cues, the taste and smell, or chemosensory, system maintains communication between the brain networks essential for emotional decisions regarding alcohol intake choices. The chemical senses primarily drive the reward value of food [7] and alcohol [8], and they play a crucial role in our overall sensory experiences and well-being [9]. The craving for alcoholic beverages is elicited by their odors as evidenced by a task-based functional MRI (fMRI) study showing an increased activation in the nucleus accumbens (NAc) and ventral tegmental areas — regions of the brain associated with reward anticipation in heavy drinkers [10]. Moreover, mesocorticolimbic activation has also been found with taste of alcohol cues [11–14]. The taste and smell of alcohol cues trigger the release of dopamine from reward centers of the brain [15], inducing craving responses. Alcohol odor cues are known to weaken inhibitory control and attentional bias as measured by the go/no-go and Stroop task [16, 17].

Despite the literature being dominated by task-based fMRI studies, recent resting state fMRI (rs-fMRI) studies have demonstrated taste and smell brain networks [18, 19]. Arnold et al. [20] examined the Human Connectome Project (HCP) S900 dataset to parcellate human olfactory networks across frontal and temporal regions by correlating 812 individual behavioral olfactory measures (i.e., odor identification) with resting-state scans using an ROI-to-ROI based approach. Further they built an optimized graph theory-based network with sensory, limbic and frontal subnetworks [20]. Moreover, gustatory information reportedly is coded by a parallelly distributed neural network [21–24]. Therefore, we propose that gustation (taste) and olfaction (smell) can be more comprehensively captured by brain-wide, large-scale interactions between distinct regions across the entire brain instead of local characteristics of a few brain regions or smaller-scale circuits. We used an approach to parcellate taste and olfactory networks utilizing the behavioral taste and odor scores with 100 nodes of resting state independent component analysis (ICA).

Prior studies have reported on the effects of short- and long-term alcohol use on whole-brain connectivity; associations of connectivity patterns with clinical manifestations of AUD [25, 26]; connectivity markers to improve prediction of AUD diagnosis [27, 28]; characterization of the frontal, parietal, subcortical and default mode networks (DMNs) connectivity patterns as a function of severity of alcohol use [29]. The absence of evidence on the association of taste and olfactory connectivity features with alcohol consumption led us to use a large-scale rs-fMRI data set to investigate the role of the functional connectome pertaining to taste and smell perception on alcohol preferences and consumption. We aimed to combine robust statistical modeling with brain connectivity patterns to predict alcohol intake behavior as previously reported by studies predicting personality traits [30–32], symptoms of attention deficit hyperactivity disorder [33] and higher-level cognitive concepts, such as creativity, in subjects that were not included in the initial analysis [34]. Building on this evidence, we aimed to determine the accuracy of predicting alcohol intake using identified taste and smell brain networks in young adults, utilizing resting state functional connectivity data from the HCP dataset. We investigated the predictive power of these connectivity patterns for individual alcohol beverage intake in study participants, hypothesizing that brain connections related to taste and smell perception are associated with alcohol intake behaviors.

## Materials and methods

### Human connectome project (HCP) data

The rs-fMRI data were acquired from the HCP Young Adult cohort (http://www.humanconnectome.org). Four resting state sessions with difference phase encoding: 2 RL and 2LR in two different days were acquired using gradient-echo echo-planar imaging (GE EPI) at 3 Tesla. The multiband EPI methodology (2mm-isotropic voxels; TR = 0.72 s) and scan length (1200 timepoints; 15 min) was used due to its high spatiotemporal resolution. For our analysis purposes, we used the HCP S1200 “PTN” (Parcellation + Timeseries + Netmats) data of 1003 study participants (all aged 22–37, *n* = 469 females), which consists of extensively processed rs-fMRI data from the HCP S1200 release. Previous publications have reported the full details regarding the sample, data acquisition and preprocessing procedures [35–37]. Participants provided written informed consent, and all procedures were approved by the Washington University Institutional Review Board (IRB# 201204036; Title: ‘Mapping the Human Connectome: Structure, Function and Heritability’). Using a random 70:30 partitioning approach we created training and validation datasets from the 1003 participants’ data to avoid over-fitting issues and to evaluate the generalizability of our findings. To ensure the comparability between the training and validation sample, comparisons were performed for sex, age, body mass index (BMI), Fagerström Test for Nicotine Dependence (FTND) score, odor identification and taste intensity scores (Welch *t*-test for continuous variables, Wilcoxon test for non-normally distributed variables, Chi-squared test for categorical variables), which are reported in the Results. Previously released “PTN” datasets have been used successfully to relate individuals’ functional connectivity data to non-imaging data (e.g., education, tobacco intake, IQ, reading ability, personality profile) [30, 38] (HCP MegaTrawl: https://db.humanconnectome.org/megatrawl/).

### Estimation of functional connectome

We utilized the time series provided in the HCP S1200 PTN data to compute individual-level functional connectomes. Specifically, the rs-fMRI data was subjected to customary pre-processing and group Independent Component Analysis (ICA) parcellation as delineated by Smith et al. [38]. To obtain individual rs-fMRI time series, a “parcellation” approach (group-ICA decomposition) was applied. This involves generating a unique time series for each ICA component by projecting the ICA spatial maps onto the rs-fMRI data of each participant. In our network analyses, we incorporated time series from 100 ICA components, treating each component as a network “node.” The node-time series were extracted using the first stage of dual-regression [38]. Here, each ICA map served as a spatial regressor in relation to the comprehensive time series data, yielding a time series for each of the 100 nodes. Each node’s time series comprised 4800 time points, collated from four sequential 15-minute rs-fMRI sessions. Subsequently, functional connectomes for each participant were constructed based on these node–time series, forming 100 × 100 matrices. Specifically, we computed the temporal correlations between the time series of nodes to facilitate further analysis of the connectomes. We created a functional connectome database that consisted of all the functional connectome (triangles without self-correlations and repeated connectivity strengths) for all the subjects within the dataset.

### Classifying ICs into signal and noise

To classify an independent component (IC) as either signal or noise, we calculated several features, including edge fraction, cerebrospinal fluid (CSF) fraction, maximum realignment parameters (RP) correlation and high-frequency content [39]. The smoothness measure assesses the contributions of low and high spatial frequency content for each IC. The edge activity measure evaluates the extent of activity in peripheral brain areas using an edge mask, while the CSF activity measure assesses activity in ventricular brain areas using a CSF mask. The temporal frequency noise (TFN) measure determines the power in temporal frequencies beyond 0.1 Hz.

We utilized the Brain Extraction Tool (BET) from the FMRIB Software Library (FSL) to isolate brain tissue from the standard MNI152 T1-weighted image, setting the fractional intensity threshold to 0.5 and adjusting the vertical gradient to 0. This process generated a brain-extracted image used for further analyses. For edge mask creation, the brain-extracted image was converted into a binary mask, which was then dilated to include a slightly larger region. Subtracting the original binary mask from the dilated mask produced the outer edge mask, highlighting the brain’s outer boundaries.

To create a CSF mask, we applied FSL’s Automated Segmentation Tool (FAST) to segment the brain-extracted image into CSF, gray matter and white matter. We then calculated the fractional volumes of different regions identified in the masks by loading the ICA spatial maps, resampling the masks to match the ICA maps’ shape.

The edge and CSF masks were used to get edge and CSF fractions, i.e., the proportion of the IC’s intensity that overlaps with the edge and CSF mask.

To calculate the maximum RP correlation, we computed the Pearson correlation between each IC time course and each motion parameter, recording the highest absolute correlation value for each IC in each subject. Additionally, the high-frequency content for each IC time course was calculated using the Welch method to estimate the power spectral density. The ratio of high-frequency power (frequencies above 0.1 Hz) to the total power was computed for each IC in each subject. The individual results were then aggregated across all subjects (mean) for each feature across ICs. Finally, using all features (maximum RP correlation, high-frequency content, edge fraction and CSF fraction) and specified thresholds (maximum RP correlation = 0.5, high-frequency content = 0.3, edge fraction = 0.4, CSF fraction = 0.3), we classified ICs as either signal or noise. Approximately forty percent of ICs were identified as signal and were considered for further analysis.

We then assigned each IC identified as signal to one of the resting-state sub-systems by first co-registering and resampling the volumetric ICA maps to the space of the 17-subsystem solution of Yeo et al. [40], available from https://surfer.nmr.mgh.harvard.edu/fswiki/CorticalParcellation_Yeo2011, using SPM12 (https://www.fil.ion.ucl.ac.uk/spm/). We calculated the spatial correlation between the ICA maps and Yeo networks to assign a network to each IC, selecting the network that showed the maximum spatial correlation [41]. The maximum spatial correlation of each IC with the respective Yeo network was greater than 0.20. Please see Supplementary File S1 shows IC-network spatial correlation of all 100 nodes.

### Measures

#### Odor identification scores

We used the Odor_Age Adjusted scores within the HCP sensory measurements. These scores were derived from participants’ performance on the National Institutes of Health Toolbox Odor Identification Test (NIHTB-OIT) [42], which is administered by a trained examiner. The test consists of nine items, each presenting a different odor on scratch-and-sniff cards. Participants are required to select the correct odor from a set of four options, which include one correct response and three distractors. The test is administered on a tablet screen, where participants view four picture and word options simultaneously in a 2 × 2 layout. The examiner reads aloud each option one at a time. Total scores range from 0 to 9, with a chance performance at 25%, approximately equivalent to a score of 2. The nine odors for identification were lemon, Play-Doh, bubble gum, chocolate, popcorn, coffee, smoke, natural gas and flowers. Additional details, such as an example response screen and examiner training materials, can be found on the NIH Toolbox website [43].

#### Taste intensity scores

We used the Taste_Age Adjusted scores within the HCP sensory measurements. Participants underwent the NIH Toolbox Regional Taste Intensity Test, which evaluates the perceived intensity of quinine (a bitter tastant), and sodium chloride (salty taste) administered in liquid solutions. These tastants are applied both to the tip of the tongue and throughout the entire mouth, and participants rate their intensity using a generalized labeled magnitude scale that spans from “no sensation at all” to “strongest sensation of any kind” [44].

#### Alcohol measures

In our study, alcohol-related variables from the HCP dataset were assessed using two instruments: the Alcohol Use 7-Day Retrospective Assessment for past-week alcohol intake and the Alcohol Use and Dependence assessment for past-year alcohol consumption. To identify if the past-week alcohol intake reflects the longer-standing patterns captured in the past-year data, we correlated the past-week and past-year alcohol intake variables. Six numerical variables were used to capture past-week alcohol intake, recorded as total alcoholic drinks consumed in the past 7 days, reported on the final day of the HCP visit. These variables included: total drinks in the past 7 days, beer/wine coolers, malt liquor, wine, hard liquor, and other alcohol. For past-year alcohol consumption, two variables were utilized: the average number of drinks consumed per drinking day over the past 12 months (SSAGA_Alc_12_Drinks_Per_Day) coded as 0, 1, 2, 3, 4, 5–6 = 5, 7 + = 6; and the maximum number of drinks consumed in a single day over the past 12 months (SSAGA_Alc_12_Max_Drinks) coded as 1–2 = 1; 3–4 = 2; 5–6 = 3; 7–8 = 4; 9–10 = 5; 11–12 = 6 if male, 5 if female; 13 + = 7, 5 if female.

### Analyses

**Fig. 1:**
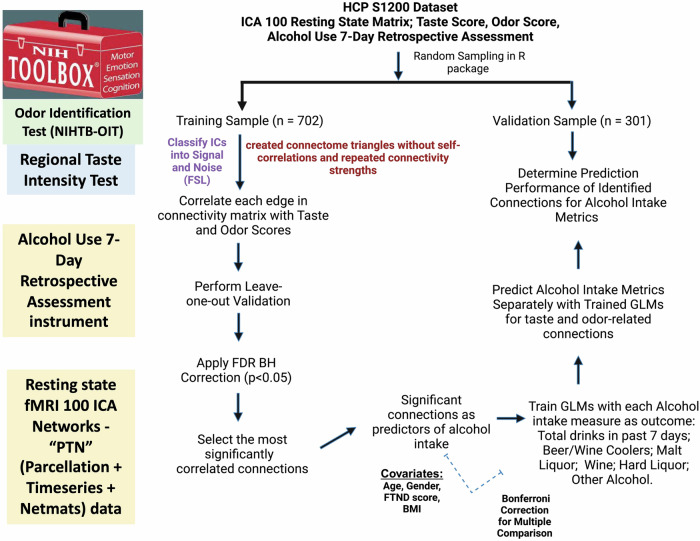
Analytical pipeline to validate brain networks for olfaction and gustation in predicting alcohol consumption. The analytic pipeline was developed to delineate brain networks associated with olfaction and gustation on a global scale and to confirm the predictive validity of these networks concerning alcohol consumption measures. Here, ICA stands for independent component analysis, FDR BH refers to false discovery rate correction using the Bonferroni-Holm method, GLM denotes the general linear model, FTND is the Fagerström Test for Nicotine Dependence, and BMI represents body mass index.

We conducted direct Pearson correlation between taste, smell measures and alcohol drinking variables (past-week). A schematic of the analytic pipeline is provided in Fig. 1 and codes are available in the Supplementary File. We imputed missing values using a proximity matrix implemented in R software, version 3.6.2. The first step was to create a training set (70% of the sample) and a validation set (30% of the sample) of participants using the caret package (topepo.github.io/caret/index.html; https://www.jstatsoft.org/article/view/v028i05) in R. This resulted in a training sample containing 702 study participants, and a validation sample containing 301 participants, excluding the missing data for any variable of interest. Followed by this step the relationship between brain connectivity patterns and chemosensory variables (odor and taste) was established, with an extension to predict alcohol consumption metrics based on these connectivity patterns. R and MATLAB scripts were written to perform the analyses described and to generate the figures presented; these codes are available upon request.

### Identification of the olfactory and gustatory network

The study employed a robust statistical framework to assess the relationship between functional connectivity strengths and odor and taste scores, separately, in an unbiased way. We performed the correlation analysis between each connection within the whole-brain functional connectome and sum scores for odor and taste scores across participants. The resulting r values were set at a threshold of *P* < 0.001. We performed a leave-one-out cross-validation approach, facilitating a comprehensive correlation analysis between individual connectivity features and chemosensory scores. Specifically, at each of the *n* iterations, one of the individuals was excluded from the analysis and assessment of Pearson correlation of odor and taste scores with each element of the connectivity matrices (Mij) was conducted. This yielded a series of *p*-values for each connectivity feature, reflecting its statistical significance in relation to odor and taste scores. To mitigate the risk of type I errors inherent in multiple comparisons, the obtained *p*-values were first averaged. Subsequently, these values underwent a False Discovery Rate (FDR) correction employing the Benjamini-Hochberg procedure (at a threshold of *P* < 0.05), ensuring the reliability of the identified significant relationships.

### Prediction and validation of related alcohol consumption measures using olfactory and gustatory networks

Matrix elements that had significant positive or negative correlations with the odor and taste scores in the training sample were identified as edges of the positive or negative adjacency matrices and used to train a general linear model (GLM) to predict each alcohol consumption metrics. The models were adjusted for potential confounders, including age, sex, BMI and FTND scores. At first we determined the association of identified significant odor and taste brain connections with alcohol intake variables (for past-year and past-week), separately. Then the significance of connectivity features in predicting alcohol consumption behaviors was assessed, and the obtained *p*-values were also adjusted for multiple testing comparisons using the Holm–Bonferroni method. Of note, these steps were conducted within the training sample for independence.

We then applied the trained GLM models (for both odor- and taste-related predictors—network connections) from the training data to our independent validation sample to estimate each model’s accuracy in predicting alcohol intake metrics (past-week). Each alcohol measure in the validation dataset was dichotomized at the median value calculated across all samples. Values above the median were coded as 1, representing “higher” alcohol consumption, while values at or below the median were coded as 0, representing “lower” alcohol consumption. Following the median split, binary classification metrics (accuracy, precision, recall, F1-score) were used to assess how effectively connectivity features and control variables predict high versus low levels of alcohol use, enhancing interpretability by framing alcohol consumption in relative terms (i.e., higher or lower than median use). Predictions were then compared with actual scores from the validation sample, and accuracy metrics including mean squared error (MSE), root mean squared error (RMSE), accuracy, precision, recall, and F1-score were calculated to evaluate the models’ performance in predicting each alcohol consumption metric.

## Results

### Comparisons between the training and validation sample

Initially, we compared age, sex, BMI, FTND scores, taste and odor scores between the training set (*n* = 702) and the matched (participants matched for odor and taste scores with those in training sample) validation set (*n* = 117). The two samples do not differ in gender (χ2 = 6.1924e–30, *P* = 1.0), age distribution (t = −0.61, *P* = 0.54), FTND scores (W = 6861.5, *P* = 0.31), taste (t = −0.64, *P* = 0.52) or odor (t = 0.01, *P* = 0.99) scores.

### Correlation of taste and smell variables with alcohol measures

Through direct Pearson correlation analysis between chemosensory variables and alcohol measures, we found a significant correlation between odor identification scores and total wine intake among young adults in our training sample. However, no statistically significant correlation was observed between odor scores and other alcohol variables. Additionally, taste intensity scores showed no significant associations with any of the alcohol measures (Please see Supplementary Table).

### Identification of the olfactory and gustatory network

**Fig. 2:**
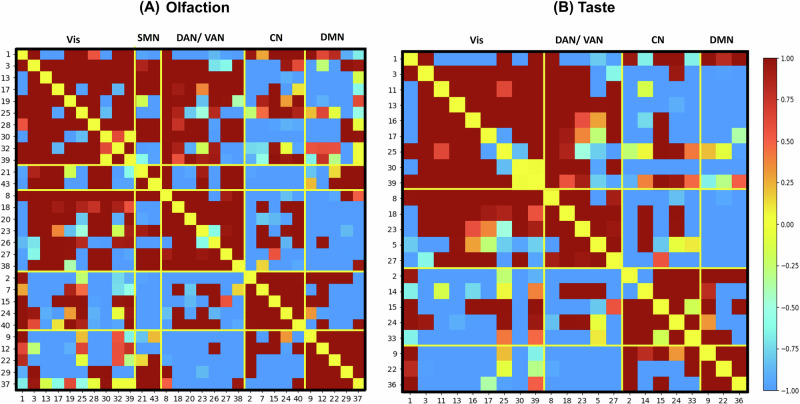
Correlation matrices of olfactory and gustatory networks reveal distinct connectivity patterns across brain regions. The correlation matrices of the computed networks for olfaction (**A**) and taste (**B**) within the training sample (primary dataset) are presented. Here, SMN stands for somatomotor network; DAN/VAN for dorsal attention network/ventral attention network; Vis for visual network; CN for control network; and DMN for default mode network. The color bar indicates the correlation coefficient, and the IC numbers are denoted on the x- and y-axes.

Out of the total 742 possible brain connections, 41 (with odor identification scores) and 25 (with taste intensity scores) connections passed the predefined statistical threshold (*P* < 0.05) during every iteration (training sample; *n* = 702) of the leave-one-out cross-validation and FDR-BH correction. One of these connections (node27-node23) was commonly associated with both odor and taste scores. The connections identified that were related with odor and taste scores have a widespread distribution across the brain and had representation in several of Yeo resting state sub-systems, viz., visual, dorsal (DAN), and ventral attention (VAN), fronto-parietal (FP)/control (CN), somatomotor (SMN), default mode networks (DMN): temporal parietal (TP) (Fig. 2).

### Olfactory and gustatory networks associated with participants’ alcohol beverage consumption

We identified a strong correlation between total drinks consumed in the past-week and number of drinks consumed per day (r = 0.45; *p* < 0.001) and the maximum number of drinks (r = 0.55; *p* < 0.0001) in the past-year. Past-week beer/wine/cooler intake was also associated with past-year measures [number of drinks consumed per day (r = 0.36; *p* < 0.0001); maximum number of drinks (r = 0.56; *p* < 0.0001)]. Wine, Hard liquor and other alcohol past-week intake were not significantly associated with past-year consumption (r = −0.01 to 0.28; *p* > 0.05).

**Fig. 3:**
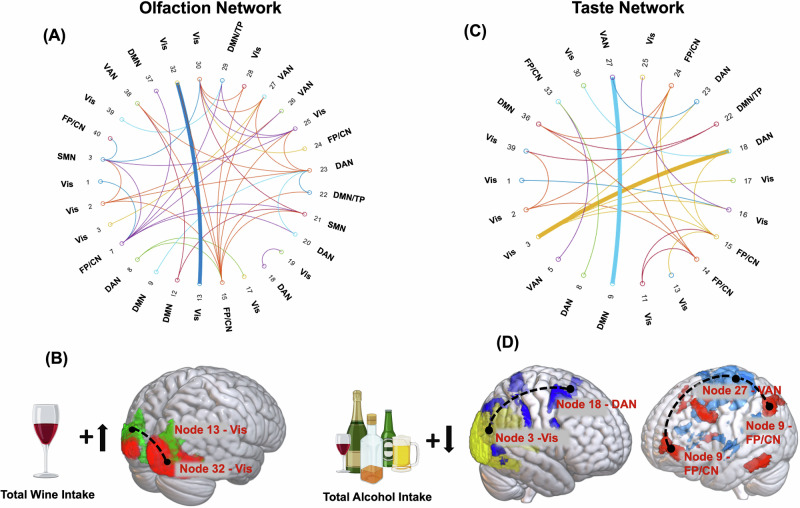
Associations between brain connectivity in olfaction and gustation networks and alcohol consumption metrics. A general linear model (GLM) was used to analyze the associations between brain connectivity variables and responses to a 7-day retrospective questionnaire for each alcohol consumption metric within the training sample. These trained GLMs were then applied to the validation sample to determine the relationship between each alcohol metric and the identified brain connections within the olfaction and gustation networks. All connections within these networks, as well as significant correlations with the alcohol consumption metrics mentioned in the manuscript, are illustrated in figures (**A**, **B**) for odor and (**C**, **D**) for taste. In the figures, an upward arrow with a plus sign indicates a positive association, while a downward arrow with a plus sign indicates a negative association between brain connections and alcohol intake metrics. Here, SMN stands for somatomotor network; DAN/VAN for dorsal attention network/ventral attention network; Vis for visual network; CN for control network; FP for frontoparietal network; DMN for default mode network; and TP for temporoparietal network.

In the adjusted and Bonferroni-corrected trained GLMs (training sample), several significant associations between the olfactory and gustatory network connections and past-week (Total drinks, Total Beer/Wine/Cooler, Total Wine) and past-year (number of drinks per day; maximum number of drinks in a single day) alcohol consumption measures were identified. Notably, within the olfactory network, there was a significant positive association between total wine consumption over seven days and the visual-visual connection (node32-node13: β = 0.028; confidence interval (CI) = 0.01, 0.05; *p*-adjusted = 0.02) (Fig. 3A, B).

In the gustation network, total alcohol intake (past-week) and maximum drinks (past-year) showed a negative association with connectivity in the VA-FP/CN [node27-node9 (past-week; β = −0.31; CI = −0.23, −0.03; *p*-adjusted = 0.04) (past-year; node27-node9: β = −0.05; CI = −0.09, −0.003; *p*-adjusted = 0.04)], while beer/wine/cooler intake (past-week) was negatively associated with connectivity between the DA-visual networks (node18-node3: β = −0.10; CI = −0.16, −0.04; *P* = 0.003) (Fig. 3C, D).

### Prediction accuracy of olfaction and gustation networks for alcohol intake

**Fig. 4:**
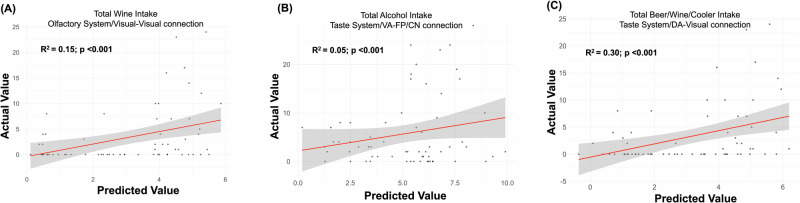
Associations between alcohol intake and brain connections. **A** Scatter plot showing the relationship between Total Wine Intake and Olfactory Network/Visual-Visual connection. A significant positive correlation is observed (R² = 0.15, *P* < 0.001), indicating a strong association between wine intake and this specific brain connection. **B** Scatter plot showing the relationship between Total Alcohol Intake and Gustatory Network/VA-FP/CN connection. The data shows a positive correlation (R² = 0.05, *P* < 0.001), suggesting a significant positive association between overall alcohol intake and this brain connection. **C** Scatter plot illustrating the relationship between Total Beer/Wine/Cooler Intake and Gustatory Network/DA-Visual connection. A moderate positive correlation is observed (R² = 0.30, *P* < 0.001), indicating a positive association between beer/wine/cooler intake and this brain connection. In all panels, the red line represents the best-fit regression line, and the shaded area around the line represents the 95% confidence interval. The actual values of the brain connections are plotted against the predicted values based on the alcohol intake measures.

The trained GLMs for both odor and taste connectivity variables were applied to the validation dataset to predict alcohol intake metrics (past-week). Scatter plots illustrate the relationship between actual and predicted alcohol intake values (past-week) for the significant connections within the olfactory and gustatory systems (Fig. 4). Accuracy variables including Mean Squared Error (MSE), Root Mean Squared Error (RMSE), accuracy, precision, recall, and F1 score were then calculated for both odor- and taste-related predictions by comparing predicted scores to actual scores from the validation data.

For odor-related connections, the model demonstrates strong predictive accuracy with a low MSE of 26.5 and a RMSE of 5.15, indicating close alignment between predicted and actual consumption values (see scatter plot Fig. 4A). The RMSE of 5.15 reflects an average prediction deviation of approximately 5.15 units. The model achieves an accuracy of 0.75, correctly predicting whether consumption is above or below the median 75% of the time. Precision (0.72) and recall (0.77) underscore the model’s efficacy in predicting higher consumption: 72% of high consumption predictions are correct, and 77% of actual high consumption instances were identified with F1 score of 0.74, balancing precision and recall. These results suggest that the connectivity between visual networks is a significant predictor of alcohol (wine) consumption, with the model performing well in terms of both prediction accuracy and high consumption identification.

For taste-related connections, the model also exhibits strong performance with a low MSE of 26.39 and an RMSE of 5.14, signifying that the predicted values closely match actual consumption (see scatter plot Fig. 4B). The RMSE of 5.14 indicates an average prediction deviation of 5.14 units. The model’s accuracy was 0.72, correctly predicting consumption above or below the median 72% of the time. Precision (0.69) and recall (0.73) reflect the model’s effectiveness in predicting higher consumption: 69% of high consumption predictions were accurate and 73% of actual high consumption instances were identified. An F1 score of 0.71 indicates a solid performance [45]. Overall, these findings demonstrate that the connectivity between VA-FP/CN is a significant predictor of alcohol consumption, with the model effectively identifying high consumption patterns for beer, wine and cooler intake over a seven-day period. Furthermore, the model shows a relatively high MSE of 50.23 and an RMSE of 7.09, indicating less accurate predictions with larger deviations for predicting total drink consumption. An RMSE of 7.09 signifies an average prediction deviation of 7.09 units. The model’s accuracy is 0.48, close to random guessing. Precision (0.41) and recall (0.48) further highlight the model’s limitations: only 41% of high consumption predictions were correct and 48% of actual high consumption instances were identified (see scatter plot Fig. 4C). An F1 score of 0.44 indicates suboptimal performance. These results suggest that the connectivity between DA-visual networks is a less accurate predictor of alcohol consumption, with the model performing poorly in terms of both accuracy and high consumption identification.

## Discussion

Our whole-brain analyses on the S1200 HCP rs-fMRI PTN dataset revealed a human olfactory and gustatory functional network of 41 and 25 interconnected parcels, respectively. Notably, within this network, three connections exhibited consistent correlations with metrics of alcohol consumption among individuals in our sample. These identified network connections showed a moderate predictive validity for alcohol consumption measures in our validation cohort. In sum, the current study provided a representative description of the human olfactory and gustatory network as a model for predicting alcohol intake behavior.

The widespread brain connections identified within the olfactory and gustatory network is consistent with the premise that the chemosensory system supports not only the olfactory and gustatory sensory perception but also multiple non-sensory functions, such as emotion, memory, attention and homeostasis (e.g., neuroendocrine regulation, feeding, etc.) [46, 47]. Numerous studies examining brain connectivity during tasks have shown that the perception of odors and tastes is enhanced in the presence of visual, emotional, learning and attentional cues, indicating correlations within the brain networks responsible for these processes. Specifically, the presence of negatively valence visual stimuli (e.g., fearful faces) and odors (e.g., aversive smells) has been found to increase positive connections between visual and emotional networks, such as the amygdala, as revealed by dynamic causal modeling (DCM) [48, 49]. Additionally, the presentation of visual stimuli has been shown to activate olfactory networks, even when presented alone; however, it’s noteworthy that these visual cues were preceded by the presentation of combined odor and visual stimuli. Furthermore, research has demonstrated connectivity between the olfactory cortex and regions crucial for associative learning, as well as in memory encoding and retrieval, using unified structural equation modeling (uSEM) [50, 51]. The impact of attention on taste perception, or gustatory processing has been investigated by studying disruptions in communication between primary and secondary taste regions in the presence of distracting cues combined with varying intensities of sweet taste cues [52]. Together these studies support our observation of the association of olfactory and gustatory perception scores with connections between visual, VA, DA, and FP/CN.

Among the connections within the olfactory and gustatory networks, positive and negative relationships with total alcohol intake, as well as total intake of wine and beer/wine/coolers in the last 7 days, were identified. Kudela et al., using a task-based dynamic functional connectivity (dFC) design in which participants were actively tasting beer or Gatorade, found increased connectivity between visual and frontoparietal (FP) and visual attention networks (VANs) specifically for beer [53]. This increased connectivity between the visual and reward processing networks likely reflects the amplified salience of rewarding alcohol chemosensory cues, which stimulate dopaminergic activity in the brain’s reward system [15, 54]. In contrast, our study employed resting-state fMRI to assess intrinsic, baseline connectivity patterns without task engagement. This fundamental difference may explain some of the observed discrepancies, as task-based studies capture immediate, stimulus-driven network activations, while resting-state studies reveal stable, intrinsic connectivity patterns. Interestingly, our findings revealed a negative association between recent wine, beer, or cooler consumption and connectivity within the visual-dorsal attention (DAN) networks. This divergence could be due to the unique sensory and cognitive processing mechanisms associated with these beverages. For instance, wine is often considered a more complex sensory experience, involving not only gustatory and olfactory perception but also texture and visual aesthetics. Variations in beverage properties, such as sugar content, carbonation, and tannin levels, may influence neural responses differently. Furthermore, the inclusion of multiple alcohol types (beer and wine coolers) in our study may contribute to our findings. This diversity of alcohol types in our analysis likely reflects a more generalized effect on connectivity rather than a taste-specific response. In addition, Kudela et al. employed a novel bootstrap-based dFC methodology that captures transient connectivity fluctuations during the tasting task. This approach is more sensitive to immediate taste- and reward-driven responses, while our static functional connectivity analysis focused on overall connectivity patterns, potentially missing these transient dynamics. This methodological distinction may also account for differences, particularly in reward and sensory networks.

The findings suggest that different types of alcoholic beverages may engage distinct neural circuits based on their sensory properties, cultural associations, and individual preferences. For instance, we did not have data on specific beer types, but a light beer with minimal aroma may elicit very different neural responses compared to a lager, IPA, or wheat beer. Similarly, while wine can vary significantly in its sensory profile from vintage to vintage, wine coolers are generally more consistent. Future studies that link these connectivity metrics with specific aspects of taste perception, especially sensitivity to bitter flavors, could provide deeper insights into how different alcohol types modulate neural circuits. In summary, differences in study design (task-based vs. rest), sample size, alcohol types, and connectivity analysis methods (dynamic vs. static) likely account for the observed discrepancies. Our results offer complementary insights, suggesting that habitual alcohol intake may influence baseline connectivity in sensory and attention networks, while Kudela et al. highlight immediate, task-evoked connectivity changes in response to alcohol taste. Together, these findings underscore the importance of employing varied methodologies to capture both acute and habitual effects of alcohol on brain connectivity.

While the regions identified in our prediction analysis within the visual and attention-control networks aren’t canonically involved in olfaction or gustation, these non-traditional regions may have stronger predictive power for behavior. Prediction models often reveal statistical associations beyond established functional roles, capturing broader network configurations that best correlate with outcomes [55]. Additionally, the ICA was entirely data-driven, avoiding biases from an a priori focus on traditional olfactory regions, as seen in many prior studies [56–58]. Such data driven approaches serve better reliability in prediction than ROI-based methods [59]. The networks identified also stem from resting-state data, which doesn’t necessarily align with task-based functional architecture but is well-suited for detecting trait-like indicators that predict behavior. Our data-driven approach identified visual-sensory and control regions as more predictive of alcohol consumption. For individuals frequently exposed to alcohol-related environments, resting-state connectivity in sensory-visual networks may reflect altered sensory perceptions. For instance, alcohol intoxication has been shown to be associated with altered visual network connectivity [60]. Additionally, control network connectivity at rest is associated with regulatory processes [58], which are crucial for predicting consumption behaviors without direct reward -alcohol cues, reflecting the intrinsic connectivity patterns. Thus, while the interplay of reward and control network is significant in addiction [61], control network outplayed reward in predicting alcohol drinking in this large dataset.

Separate prediction models were set up for each alcohol intake metric to estimate the prediction accuracy of the olfaction- and gustation-related network connections. The model shows strong accuracy for odor-related connections, with an MSE of 26.5, RMSE of 5.15, and an accuracy of 75%. Precision is 0.72, recall 0.77, and the F1 score is 0.74, indicating moderate performance and highlighting the significance of visual network connections in predicting wine consumption. However, the relatively modest R-squared value of 0.15 (*p* < 0.001) indicates that while there is a meaningful association, only a small proportion of the variance in wine consumption is explained by these connections, likely due to the complexity of factors influencing alcohol intake. For taste-related connections, the model also performs well, with an MSE of 26.39, RMSE of 5.14, 72% accuracy, precision at 0.69, recall at 0.73, and an F1 score of 0.71. The connectivity between VA-FP/CN is a significant predictor of alcohol consumption (beer, wine, and cooler intake), achieving an R-squared of 0.30 (*p* < 0.001). This value suggests a somewhat stronger relationship, though the model still captures only part of the overall variation in these intake patterns. In predicting total drink consumption, however, the model is less effective, with an MSE of 50.23, RMSE of 7.09, 48% accuracy, precision at 0.41, recall at 0.48, and an F1 score of 0.44. The low R-squared value of 0.05 (*p* < 0.001) further indicates large prediction deviations and highlights the limitations of using connectivity metrics alone to predict total alcohol intake.

These findings highlight the complexity of the neural mechanisms underlying alcohol consumption and emphasize the need to consider multiple factors such as individual differences in taste and odor preferences, genetic predispositions, environmental influences, psychological factors, and social contexts when developing predictive models. Our study also suggests that brain connectivity related to taste and olfactory perception may serve as predictors of alcohol consumption in young adults under typical conditions. During the pandemic, heightened stress, social isolation, and other psychological pressures likely contributed to increased alcohol consumption as a coping mechanism, irrespective of sensory input. This indicates that while sensory processing and associated brain connectivity can be reliable predictors of alcohol intake, psychological and contextual factors such as stress, anxiety, and disrupted routines can significantly influence drinking behaviors. Further research could explore the specific neural circuits and mechanisms that mediate the relationship between connectivity within these networks and various patterns of alcohol consumption.

The present work has a few limitations worth noting. Although our sample size is large and the brain connections demonstrated prediction accuracy for alcohol intake in our in-built validation cohort, replication of findings in an external dataset would be crucial to strengthen the generalizability of these results. The use of a cross-sectional design to investigate chemosensory-connectivity associations limits our ability to infer causality or rule out cohort effects. A longitudinal design could provide greater insight into the temporal relationships and causative pathways underlying these associations. Additionally, it is possible that intermediate phenotypes, such as personality traits or neurocognitive performance, may mediate the relationship between brain networks and alcohol consumption behavior. However, this analysis focused specifically on the predictive value of brain networks associated with odor and taste sensitivity for alcohol intake behaviors. Future studies could explore the mediating effects of emotional states on the link between brain connectivity and alcohol consumption, which may further elucidate these associations. Moreover, genetic markers, such as PROP (6-n-propylthiouracil) supertaster status, may partially explain some of the observed findings, warranting investigation in other population datasets. Although unlikely, variations in scan length across participants could have introduced subtle effects on some connectivity metrics, which should be examined in future analyses. Addressing these factors will help refine our understanding of how chemosensory brain networks relate to alcohol intake behaviors and the broader implications for neurobehavioral health.

## Conclusion

In summary, our findings advance those of previous studies showing associations among gustatory and olfactory function scores and within‐networks connectivity. We identified strong and specific associations between chemosensory percepts (odor identification and taste intensity) and various brain connections, including those within the visual, DAN, VAN, SMN, FP/CN, and DM networks. Notably, we demonstrated that connections within the olfactory and gustatory networks can accurately predict alcohol intake metrics in young adults. Future studies should aim to include a broader age range to further explore these associations.

## Supplementary information

Correlation of odor and taste scores with alcohol drinking variables.

IC-network spatial correlation of all 100 nodes.
