# Supplementary material for: Prediction of alcohol intake patterns with olfactory and gustatory brain connectivity networks
Source: Neuropsychopharmacology. 2025 Feb 17;50(7):1167–75. doi: 10.1038/s41386-025-02058-7 (PMC12089591; doi:10.1038/s41386-025-02058-7)
Supplement: Supplementary file 1 — Correlation of odor and taste scores with alcohol drinking variables. [file 41386_2025_2058_MOESM1_ESM.docx]

Supplemental Table | Correlation of odor and taste scores with alcohol drinking variables

| ***Taste scores*** | **Correlation Coefficient** | **P value** |
| --- | --- | --- |
| Total Beer/Wine/Cooler 7days | -0.05 | 0.23 |
| Total Malt/Liquor 7days | 0.003 | 0.92 |
| Total Wine 7days | -0.04 | 0.65 |
| Total Hard Liquor 7days | -0.02 | 0.57 |
| Total Other Alcoholic Beverages 7 days | 0.02 | 0.66 |
| Total Drinks 7 days | -0.05 | 0.17 |
| ***Odor scores*** |  |  |
| Total Beer/Wine/Cooler 7days | -0.03 | 0.38 |
| Total Malt/Liquor 7days | -0.01 | 0.77 |
| Total Wine 7days | 0.10 | **0.01*** |
| Total Hard Liquor 7days | -0.05 | 0.21 |
| Total Other Alcoholic Beverages 7 days | -0.01 | 0.79 |
| Total Drinks 7 days | -0.02 | 0.57 |

**Note:** Here * indicates significance with p-value<0.05.
